# Multiple dimensions of stress vs. genetic effects on depression

**DOI:** 10.1038/s41398-021-01369-9

**Published:** 2021-04-29

**Authors:** Mark D. Kvarta, Heather A. Bruce, Joshua Chiappelli, Stephanie M. Hare, Eric L. Goldwaser, Jessica Sewell, Hemalatha Sampath, Samantha Lightner, Wyatt Marshall, Kathryn Hatch, Elizabeth Humphries, Seth Ament, Alan R. Shuldiner, Braxton D. Mitchell, Francis J. McMahon, Peter Kochunov, L. Elliot Hong

**Affiliations:** 1 Maryland Psychiatric Research Center, Department of Psychiatry, , University of Maryland School of Medicine, Baltimore, MD 21201 USA; 2 Institute for Genome Sciences, Department of Epidemiology and Public Health, University of Maryland School of Medicine, Baltimore, MD 21201 USA; 3 Department of Medicine, University of Maryland School of Medicine, Baltimore, MD 21201 USA; 4 Human Genetics Branch, National Institute of Mental Health Intramural Research Program, Bethesda, MD 20892 USA

**Keywords:** Depression, Clinical genetics

## Abstract

Many psychiatric disorders including depression involve complex interactions of genetics and environmental stressors. Environmental influence is challenging to measure objectively and account for in genetic studies because the necessary large population samples in these studies involve individuals with varying cultures and life experiences, clouding genetic findings. In a unique population with relative sociocultural homogeneity and a narrower range of types of stress experiences, we quantitatively assessed multiple stress dimensions and measured their potential influence in biasing the heritability estimate of depression. We quantified depressive symptoms, major lifetime stressors, current perceived stress, and a culturally specific community stress measure in individuals with depression-related diagnoses and community controls in Old Order Amish and Mennonite populations. Results showed that lifetime stressors measured by lifetime stressor inventory (*R*^2 ^= 0.06, *p* = 2 × 10^−5^) and current stress measured by Perceived Stress Scale (*R*^2 ^= 0.13, *p* < 1 × 10^−6^) were both associated with current depressive symptoms quantified by Beck Depression Inventory in community controls, but current stress was the only measure associated with current depressive symptoms in individuals with a depression diagnosis, and to a greater degree (*R*^2^ = 0.41, *p* < 1 × 10^−6^). A novel, culturally specific community stress measure demonstrated internal reliability and was associated with current stress but was not significantly related to depression. Heritability (*h*^2^) for depression diagnosis (0.46 ± 0.14) and quantitative depression severity as measured by Beck Depression Inventory (0.45 ± 0.12) were significant, but *h*^2^ for depression diagnosis decreased to 0.25 ± 0.14 once stressors were accounted for in the model. This quantifies and demonstrates the importance of accounting for environmental influence in reducing phenotypic heterogeneity of depression and improving the power and replicability of genetic association findings that can be better translated to patient groups.

## Introduction

Depression is a leading cause of morbidity and mortality worldwide^1^. There has been great interest in the search for its genetic predisposition as depression is heritable, in that a proportion of its phenotypic trait is due to genotypic variation. Estimates for the heritability (*h)*^2^ of depression range from 8.7% to above 40% based on different study designs^2,3^. Efforts to identify the genetic determinants of depression have intensified with the continued emergence of large datasets available for genome-wide studies such as 23andMe and the UK Biobank^2,4^. These data support a strong genetic basis for depression, but the relatively low *h*^2^ indicates that a majority of the variance of depression is from environmental factors, which are often unaccounted for in most genetic studies. The clearest environmental etiological factor associated with depression is stress, with an incidence odds ratio of up to 5.6 in the month of a stressful life event^5^. The evidence of environmental stress that contributes to depressive symptoms has led to a stress-diathesis model of depression, with stress acting upon genetic vulnerability to cause depressive symptoms^6,7^. Most major genome-wide studies of depression searching for candidate genes do not include stress measures in their phenotypic definition^2,4,8,9^, despite indications that incorporation of stressful life events and their interaction with genetic susceptibility may improve the explanatory power towards depression^10^, and limited depression phenotyping without accounting for environmental stressors may contribute to reduced specificity of findings for depression^11^. Attempts to improve detection of genes with small effects on depression have primarily consisted of further increasing sample sizes or attempting to refine the depression phenotypes^9,12^ while accounting for gene x stressor interaction is often limited by insufficient or lack of environmental assessments in large sample studies^13^. Genetic etiology studies for depression will likely benefit from a greater understanding of the interactions of genetics and environmental stressors^14–17^. Here, we aimed to provide an estimate of the extent to which stress measures may confound genetic heritability measures of depression in a population with relative socioeconomic homogeneity.

Stress is a broad term and is used in many contexts. Stress may refer to deleterious physiological effects directly enacted upon the individual by medical disease, toxin exposure, oxidative reactions, etc., although psychological and psychosocial stress specifically is highly implicated in depression onset and recurrence^18,19^. The heterogeneous basis of depression is also complicated by varying effects of and responses to stress that can be adaptive or harmful^20^. The multitude of chronic and acute effects of stress exposure throughout life on vulnerability versus resilience to depression can further compound the phenotyping effort for depression genetic studies^21–26^. In this study, we aimed to estimate the heritability of depression by accounting for three dimensions of stress exposure—lifetime stressor exposure, currently perceived stress, and community-specific sociocultural stress—and then study their potentially unique contributions to depression in a model population in which stressors can be more precisely captured.

The Old Order Amish and Mennonite (OOA/M) populations are unique in terms of relatively limited genetic diversity, stemming from a bottleneck event as a relatively small number of Amish and Mennonites immigrated from Europe and settled in Lancaster, Pennsylvania. The geographically confined community, relative uniformity in educational background, socioeconomic status, and reduced influence by alcohol and illicit drugs, combined with large family sizes, make the OOA/M a powerful population sample to study the relative genetic and environmental contributions to depression with substantially reduced confounds^27–29^.

To the best of our knowledge, no previous studies have attempted to assess multiple dimensions of stress jointly and evaluate their potential impact on assessing genetic contributions to depression, despite this as an increasingly known confound for genetic studies, and an increasingly important approach to meeting the clearest challenges in novel genetic studies of depression^30^. In the present study, lifetime stressor exposure and current state stress were examined using existing tools^31,32^. Community and culture-specific stress are difficult to measure across the population, and thus their impacts on depression are poorly understood. In the OOA/M, the well-defined social and religious norms^33^ provide an unusual opportunity (i) to quantify life stress experiences more precisely than in the general population with reduced “noise” and (ii) to study whether culturally specific stress may contribute to depression or modify the estimated genetic heritability of depression. We thus developed a questionnaire to detect potential stress-promoting or stress-protective effects associated with feelings of nonconformity to the social and religious norms in the OOA/M. We hypothesized that discordant feelings within the community may contribute towards depressive symptoms as an independent stressor. Together, we examined the effects of three stress measures (lifetime, current, and community conformity) on current depressive symptoms, and illustrated their impact on influencing the estimated genetic components of depression.

## Methods

### Subjects

Old Order Amish and Mennonites (OOA/M) from Pennsylvania and Maryland with large family pedigrees were recruited as part of the Amish Connectome Project. Participants were recruited if at least two 1st degree family members have a psychiatric disorder and family members with and without psychiatric diagnoses were recruited. Trained clinicians conducted Structured Clinical Interview (SCID) for DSM-IV or DSM-V to establish the presence or absence of a psychiatric diagnosis, and the diagnosis was reviewed in consensus meetings. Families without psychiatric illnesses were also recruited as controls. Exclusion criteria included the history of epilepsy, cerebrovascular accident, head injury with cognitive sequelae, intellectual disability, and unstable major medical conditions at the time of the study. Research participants with substance dependence within the past 6 months or current substance use disorder (except nicotine) were also excluded. The sample included 120 participants with a current or past diagnosis of clinical depression (depression-related disorder, DRD) and 319 community controls with no psychiatric diagnosis (CC). The DRD cases included mostly those who met criteria for Major Depressive Disorder (single episode and recurrent) (*n* = 109, with 21 or 19.3% with a current active episode at the time of the study) but also included individuals with Other Specified Depressive Disorder (*n* = 6), Persistent Depressive Disorder (*n* = 4), and Adjustment Disorder with Depressed mood (*n* = 1). Individuals with bipolar disorder were excluded from this study. CC were individuals without a DSM diagnosis. All participants gave written informed consent approved by the University of Maryland Baltimore IRB.

### Quantitative depression and stress measures

#### Beck depression inventory (BDI)

A self-reported 21-item Beck Depression Inventory questionnaire rates the severity of symptoms within the recent two weeks^34^, with scores ranging from 0 to a maximum of 63. 437/439 participants completed this inventory.

#### Life stressor inventory (LSI)

To assess lifetime stress we adapted items from the Life Stressor Checklist–Revised^32^ to assess by self-report stressful events over the lifetime, for example, experiences of abuse, neglect, or violence. The total score is based on counts of reported events, ranging from 0 to 15. 418/439 participants completed this inventory.

#### Amish community stressor survey (ACSS)

Potential stress from an OOA/M-specific cultural and religious lifestyle was assessed using a novel 15-item questionnaire developed to evaluate feelings of belonging vs. nonconformity in this community (Supp. Table 1). Examples of questions include “I fit in well with my Plain Church community” and “I feel as though following church and community rules restricts my personal desires and values” with responses were given on a 5-point Likert scale from “strongly disagree” to “strongly agree.” Individual items with a positive valence in feeling were scored in reverse while negatively balanced items were scored as is so that high scores represented higher levels of reported community stress or feeling of non-conformity. Scores range from 0 to a max of 60. 433/439 participants completed this survey.

#### Perceived stress scale (PSS)

To assess the current subjective stress levels we used the self-report PSS, one of the most widely used instruments quantifying the perception of stress. It asks the participant to rate the frequency of certain stress-related feelings over the last month, i.e., feeling overwhelmed versus feeling confident in the ability to cope with problems^31^. All 439 participants completed this survey.

### Genotyping and statistics

Heritability is defined as the ratio of variation in genetic makeup to the variation of phenotypic makeup, that is, the proportion of phenotypic or behavioral differences that can be explained by genetics. Traditional heritability studies rely on the self-reported family relationship of the participants. Using thousands of single nucleotide polymorphisms one can establish an SNP-based genetic relatedness amongst participants that provide more precise familial relatedness information among the participants^35^ especially in samples with the complex familial relationships as in a population isolate like the Amish. One can then use the genetically established kinship matrix to calculate the heritability of the depression phenotypes^35^.

Genotypes were available in 401 individuals, completed using Illumina Infinium Global Screening Array v2.0 SNP-array that provides coverage for 613,599 polymorphic markers selected to provide high imputation accuracy for population-scale genetics studies. The 407,171 SNPs that satisfied the ENIGMA protocol’s quality control criteria were used for quantification of the degree of relatedness and imputation. These procedures were the same as used for Human Connectome Project subjects. Empirical kinship was generated by quantifying the similarity in the whole genome among the study participants. The coefficient of relationship using the correlation coefficient among the allelic scores weighted by the minor allele frequency factor (or Weighted Allelic Correlation, WAC-1 approach) was used to generate empirical kinship values^35^.

Genetic contribution to depression was evaluated by heritability, reflecting the proportion of phenotypic variance attributed to additive genetic effects. Heritability estimates were calculated for depressive symptoms based on BDI, as well as depression diagnosis. We estimated heritability using the variance components analysis method implemented with Sequential Oligogenic Linkage Analysis Routines or SOLAR-Eclipse software v8.5.1^36^. The total variance of a phenotype was partitioned into a genetic component due to additive polygenic effects (*h*^2^), estimated shared environmental effects based on currently shared household dwelling (*c*^2^), and a random environmental component. SOLAR-Eclipse’s heritability calculations are accelerated using the Fast Permutation Heritability Inference (FPHI) approach. As part of the larger Amish Connectome Project, we did not specifically power the study for this *h*^2^ analysis but for other purposes. Using data from a recent study by our research team in the same population with the same kinship matrix^37^, we used the SOLAR-Eclipse “h2power” command, yielding that, without including covariates, detecting a heritability of ~0.20 at *p* = 0.05 with 80% power required sample size of *n* = 326.

Sex and age were used as covariates in all analyses including *h*^*2*^ and their effects noted were significant. SPSS software version 25 (IBM Corp., Armonk, NY) was used for correlational analyses and for parametric statistical analysis including multiple regression analysis and *t*-tests for group comparisons. In all parametric analyses, the variance was not significantly different between groups being statistically compared (*p* > 0.05 for tests for normality in *t*-tests, *p* > 0.05 for heteroscedasticity in F-tests), and the assumptions for these tests including normality were met. All reported center values are means with standard deviation. Error bars shown in figures are standard deviation (SD).

## Results

### Population sample characteristics and stress measures

**Fig. 1:**
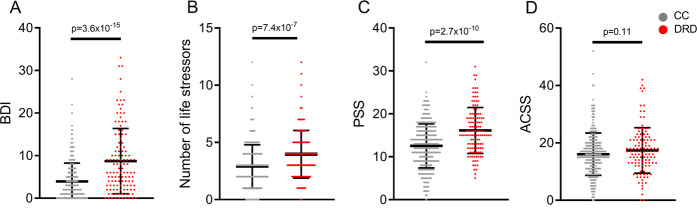
Depression and stress measures of depression-related disorder cases (DRD) compared to community controls (CC). Error bar: SD. From left to right: (**A**) Beck Depression Inventory (BDI), (**B**) number of life stressors, and (**C**) Perceived Stress Scale (PSS) were all significantly higher in DRD cases compared to controls, while (**D**) community stress measured by Amish Community Stressor Survey (ACSS) was not significantly different. *P*-values are shown based on two-tailed *t*-tests.

The sample included 439 participants (male/female ratio: 179/260) with an average age of 40.9 ± 18.0 years (age range 12 to 81). Mean age did not significantly differ between DRD and CC (*t* = 1.33, *p* = 0.19). A non-significantly higher proportion of females were in DRD (82/120 = 68.3%) than CC (178/319 = 55.8%; *χ*^2^ = 5.7, *p* = 0.17). Compared to CC, the DRD participants reported a significantly higher severity of depression symptoms by BDI (8.7 ± 7.7 vs. CC 4.0 ± 4.3, *p* = 3.6 × 10^−15^), number of lifetime stressors by LSI (4.0 ± 2.1 vs. CC 2.9 ± 1.9, *p* = 7.4 × 10^−7^), and current perceived stress level by PSS (16.1 ± 5.4 vs. CC 12.6 ± 5.1, *p* = 2.7 × 10^−10^), but reported no significant elevation in community and culturally-specific stress as measured by the ACSS (*p* = 0.11, Fig. 1). As the ACSS is a novel questionnaire, we evaluated reliability, face validity, and internal structure of the questionnaire, detailed in Supplemental Material, Appendix 1, Fig. S1, Tables S2–S4. Essentially, ACSS showed good test-retest reliability in a subset of participants who repeated the survey after 2.8 ± 0.4 years with intraclass correlation coefficient (ICC) = 0.89, (*p* = 3 × 10^−7^). The scale also showed several expected properties including that higher ACSS scores were correlated inversely with older age (*r* = −0.16, *p* = 0.0002) and positively with higher current perceived stress in the combined group (*r* = 0.17 *p* = 0.001). Additional validation analyses are available in Supplemental Material.

### Relationships between stress measures and current depression

**Fig. 2:**
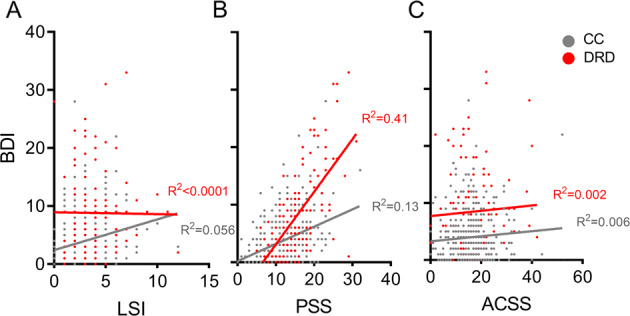
Relationship between current depressive symptoms and three different stress measures. All *R*^2^ values determined by linear regression, with p-values determined from Pearson’s *r*. **A** There was a significant but weak correlation between number of life stressors and BDI in CC (*p* = 0.00003), but not DRD (*p* = 0.93). **B** Current stress measured by PSS was positively correlated with BDI in both groups (*p* < 1 × 10^−6^), with a greater effect in DRD. **C** Neither group exhibited a significant correlation between community stress measured by ACSS and depressive symptoms (DRD *p* = 0.63, CC *p* = 0.17).

There was a weak but significant correlation between number of life stressors and BDI in CC (*R*^2^ = 0.06, *p* = 0.00003), but not among those with a depression diagnosis (*p* = 0.93, Fig. 2). Current stress measured by PSS was positively correlated with BDI in both the DRD (*R*^2^ = 0.41, *p* < 1 × 10^−6^) and the CC group (*R*^2^ = 0.13, *p* < 1 × 10^−6^); the effects were significantly stronger in DRD (*z* = 3.58, *p* = 0.0003). In neither group was community stress measured by ACSS correlated with depressive symptoms (*p* > 0.16 for both).

Multiple regression analysis was performed to examine how the three stress measures, sex, and age contributed to depression symptoms measured by BDI in each group. In DRD, the regression model was significant (F(5,107) = 18.2, *p* = 5.1 × 10^−13^) and only current perceived stress measured by PSS was a significant predictor (*t* = 9.35, *p* = 1.5 × 10^−15^). In community controls, the model was also significant (F(5,292)=12.6, *p* = 4.0 × 10^−11^) and lifetime stress (*t* = 2.95, *p* = 0.0035) and current perceived stress (*t* = 6.4, *p* = 7.3 × 10^−10^) were significant predictors. Collinearity analyses showed that multicollinearity was not a concern in the two models (all VIFs <1.25).

### Heritability of depression phenotypes

The *h*^2^ for quantitative (BDI) and discrete categorical (diagnosis) depression measures differed significantly from zero at 0.45 ± 0.12 (*p* = 7.0 × 10^−5^) and 0.46 ± 0.14 (*p* = 3.4 × 10^−3^), respectively. We then tested the hypothesis that accounting for the significant stress measures would impact heritability estimates of depression. Indeed, including both PSS and LSI totals as covariates decreased the heritability of both depression measures (Table 1, bottom), more pronounced for categorical depression (*h*^*2*^ difference −0.21) and less so for quantitative depression (*h*^*2*^ difference −0.04); however, the heritability of both remained significant, suggesting that stress may account for part of the presumed genetic contributions to depression. We thus considered whether these stress measures themselves were heritable.

**Table 1:** Heritability estimates of depression measures.

	Quantitative depression (BDI) (*n* = 369)	Categorical depression (Diagnosis) (*n* = 371)
*h*^2^	0.45 ± 0.12 (*p* = 6.97 × 10^−5^)	0.46 ± 0.14 (*p* = 0.0034)
*h*^2^ (stressors covaried)	0.41 ± 0.13 (*p* = 0.00027)	0.25 ± 0.14 (*p* = 0.024)

All were covaried with age and sex. In the bottom row, current (PSS), and lifetime stress (LSI) measures were used as covariates as well, leading to decreased, but still significant, heritability estimates.

### Heritability of stress measures

*h*^2^ for current perceived stress as measured by PSS did not differ significantly from zero (0.17 ± 0.18, *p* = 0.18, see Table 2), and neither was number of life stressors *h*^2^ (0.19 ± 0.14, *p* = 0.08). Surprisingly, the community-specific stressor measured by ACSS was highly heritable (*h*^2^ = 0.67 ± 0.10, 1.2 × 10^−8^). We suspected that this may be due to specific shared environmental effects through household dwelling level correlations. Individuals sharing the same address were counted as from the same household. There were 131 households with at least two individuals (range 2–8 individuals/house) and an additional 130 singletons. Using this approach, the household shared environmental effect (*c*^*2*^) for ACSS was significant at 0.20 ± 0.09 (*p* = 0.02) while *h*^2^ was reduced to 0.38 ± 0.14 (*p* = 0.02). *c*^*2*^ was also significant for PSS (0.15 ± 0.09, *p* = 0.03), but not number of life stressors (0.09 ± 0.09, *p* = 0.13).

**Table 2:** Heritability estimates of stress measures.

	Current stress (*n* = 371)	Lifetime stress (*n* = 353)	Community specific stress (*n* = 367)
*h*^2^	0.17 ± 0.18 (*p* = 0.18)	0.19 ± 0.14 (*p* = 0.08)	0.67 ± 0.10 (*p* = 1.2 × 10^-8^)
*h*^2^ with *c*^*2*^ included	0.14 ± 0.19 (*p* = 0.2)	0.10 ± 0.14 (*p* = 0.2)	0.38 ± 0.14 (*p* = 0.02)
*c*^*2*^	0.15 ± 0.09 (*p* = 0.03)	0.09 ± 0.09 (*p* = 0.13)	0.20 ± 0.09 (*p* = 0.02)

All were covaried with age and sex. Current stress based on PSS. Life stress based on number of life stressors in LSI. Community-specific stress based on ACSS. *n* varied slightly as not all individuals had completed all questionnaires.

## Discussion

In this study, we tested the effect of three separate dimensions of stress measures on current depressive symptoms in a unique population isolate in which environmental influence is unusually estimable and provide an illustration of the confounding that different lifetime and current stressors may have on depression heritability estimates. For community controls, both major lifetime stressors and current stress contributed modestly to current quantitative depression scores. For participants with a depression diagnosis, only the current stress contributed to current depression scores. Without accounting for stress measures, we estimated the phenotypic variance accounted for by genetic difference or heritability for quantitative and categorical depression diagnoses was similar at *h*^*2*^ = 0.45–0.46. This heritability estimate for categorical depression dropped to 0.25 once significant stress measures were accounted for.

The *h*^*2*^ estimate of 0.45–0.46 is on the upper end of other estimates, including twin studies^3,38^. The decrease in the heritability for categorical depression once stress measures were incorporated was counterintuitive, as we predicted that adjustment for environmental confounds should increase the *h*^2^ estimate for depression. This instead suggests that the estimate of genetic effects on depression may not be entirely due to genetics per se, but the interaction of genetics with stress dimensions, especially since the stressor variables were not highly heritable themselves. An alternative explanation is that including this environmental heritability factor may reduce heritability variance from the full model, leaving less variance for genetics to explain. Determining and understanding the role of gene*environment interactions is an increasingly important facet of depression^30^. Examining the interaction of stress exposure with genetics could be a powerful tool towards improving our ability to predict vulnerability to vs. resilience against depression under current stress. Recent data have shown that genetics measured by polygenic risk score for MDD derived from large population datasets better predicted depression under stress than depression at baseline in a longitudinal cohort^39^. These findings together suggest that capturing stress as part of depression phenotyping is key to obtaining more robust genetics association results.

The current study utilized quantitative depression scores that give the opportunity to capture subclinical symptoms and their potential contributions to the genetic basis for depression. Quantitative depression scores for current symptoms were higher amongst the DRD group, even though most individuals in the DRD group were in remission. The heritability analysis demonstrated that quantitative depression was less affected by stress measures than a categorical depression diagnosis. Incorporation of environmental factors would be expected to decrease the variance explained by the respective depression traits in parallel if they held the same relationship with these factors, but this was not the case as the heritability for quantitative depression remained largely unchanged. The reason for this is not clear and maybe in part due to differences in resolution (binary vs. a continuum), but our findings suggest that stress may differentially influence the assessment of quantitative vs. categorical depression phenotypes and their associations with the underlying genetics.

We observed an increased number of reported lifetime major stressors and current perceived stress amongst those who had a depression-related diagnosis, consistent with past studies^5,40,41^. The intriguing finding that those with a depression-related diagnosis had higher lifetime stress but that this was not a driver of current symptoms suggests that the cumulative effect of major stress may make an individual more likely to develop clinical depression over time but does not necessarily drive current symptoms. Environmental stressors are not normally incorporated in most genetic studies of major depression, in part because of the challenge of operational definition, as stress is a multifaceted, complex concept involving, but not limited to, lifetime cumulative stressors, community or culturally specific stressors, and current ongoing stressors. Such unaccounted-for environmental stressors, as well as resiliency factors and events, may contribute to the wide range of the reported heritability estimates and the difficulty in replicating genetic association findings in depression. Biopsychosocial contributions to depression in human studies demand a proper account of such environmental stressors to establish a valid genetic-environmental model for major depression.

In our sample population, overall rates for diagnosis of lifetime major depression (25.8%) were on the higher end of general population estimates which generally range from about 11–21.0%, but can be as high as ~28% in the U.S. general population^42,43^. Enrichment in the number of cases compared to controls may be due to two factors: recruitment methods for the Amish Connectome Project inviting large families with at least 2 DSM diagnoses, and a higher proportion of females participating making up nearly 60% of the study. Indeed, a higher depression symptom burden was found amongst females, as has been widely reported previously^24,44^.

In addition to lifetime and current stress, in this study, we examined culturally specific stress, an exceedingly difficult task in larger cohort studies in the general population. While lifetime and current stress measures were not significantly heritable as expected, the novel community-specific stressor assessment was highly heritable, although about half of the estimate could be due to household effects *c*^*2*^. We did not account for the potential past shared developmental environment, which may further increase *c*^*2*^^45^.

The ACSS is a novel survey. Preliminary analysis demonstrated good test-retest reliability 2 to 3 years apart and showed strong internal consistency (details in Supplemental Material). The scale did not significantly correlate with lifetime stressors, suggesting specificity as a community-level stress factor should not be significantly impacted by past individual-level stressors. A modest positive correlation with perceived stress in the most recent two weeks as measured by PSS (*r* = 0.17, *p* = 0.001) supports that ACSS captures some aspects of recent stressful experiences. Finally, ACSS also showed a significant inverse correlation with age, which is expected as increases in conformity to community norms and life satisfaction are common as one ages^46^. Together, these findings provide initial evidence of the validity of the ACSS. The ACSS is broadly similar to other attempted measures of the psychology of belonging, a nascent but increasingly studied dimension that has been associated with social participation and resilience factors that have implications in depression across diverse populations^47–50^. Interestingly, there was no significant association between ACSS and depressive scores in either the case or control group. We believe that this is an important finding, suggesting that the strict OOA/M community and religious lifestyle, at least as perceived by outsiders, is likely associated with important community functioning and not necessarily a psychological contributor to depression. Classical psychological literature suggests that individuals compare themselves with those of similar station, education, and background, which would allow for psychological mitigation of stressors if it were perceived that others in the same community were also exposed to the same perceived stressors^51^. There are additional potential explanations that would require further sociological and more in-depth psychological studies.

There are several limitations to the present study. The direction(s) of causality between stress and depression cannot be determined by this study. Longitudinal studies capturing a more precise onset of clinical depression in relationship to stress exposure would be critical. The OOA/M lifestyle may limit generalizability but at the same time presents valuable opportunities for the study of disease mechanisms due to reduced confounds, perhaps analogous to the rationale for using laboratory systems to study disease mechanisms under more controlled experimental environments. Studies in founder populations with defined sociocultural and community boundaries such as the OOA/M provide an opportunity to detect genetic associations with substantially increased power and also allows for careful study of environmental contributions^52,53^. The ACSS scale was developed with a Westernized or “English” viewpoint on nonconformity as potential stressors, whereas the inverse could be true for many individuals in OOA/M communities. Finally, the lifetime stressor assessments were retrospectively collected, and our three stress assessments may yet miss other important elements of the multiple dimensions of stress experienced across a lifetime associated with depression, including perceived controllability of stress versus unpredictability, as well as any effect of depression on recall bias of stressful events.

In conclusion, this study highlights the multivariate effects of stress on depression phenotyping and the potentially significant impact on heritability estimates, and thus genetic association studies of depression and our understanding of gene, environment, and gene*environment interactions in etiology of patient symptoms. Phenotyping efforts for genetic studies of depression may be more powerful by considering the role of stressor effects and the dynamic interaction with depressive symptoms. We posit that future genetic studies of depression should increasingly account for a measurable lifetime and current stress measures and examine how different stressors may have differential effects in cases and controls. These results invite further efforts to improve both animal models and human studies aiming to elucidate the etiology of depressive symptoms through a better understanding of interactive genetic and stress contributions.

## Supplementary information

Supplemental Material

## Data Availability

SOLAR-Eclipse software v8.5.1 is freely available at http://www.solar-eclipse-genetics.org. Legacy versions or code for specific analysis or the software, in general, is available by request (pkochunov@som.umaryland.edu).
