# Supplementary material for: Multiple dimensions of stress vs. genetic effects on depression
Source: Transl Psychiatry. 2021 Apr 29;11:254. doi: 10.1038/s41398-021-01369-9 (PMC8085217; doi:10.1038/s41398-021-01369-9)
Supplement: Supplementary file 1 — Supplemental Material [file 41398_2021_1369_MOESM1_ESM.docx]

**Supplemental Material**

**Supplemental Table 1**: Amish Community Stressor Survey (ACSS)

*Instructions to participant:* The following questions intend to assess community life specific to the Amish community. We are interested in the aspects of Amish life that you may find helpful, as well as things that you may find stressful. There are absolutely no right or wrong answers. As in all the studies, answering these questions is voluntary; you do not have to complete this form. However, if you can answer the questions, it would make this research study more worthwhile.

*Replies to each statement range from 0-Strongly Disagree to 4-Strongly Agree*

| 1. I fit in well with my Amish church community. |
| --- |
| 2. I have felt that I needed to force myself to go to church activities. |
| 3. I am willing to give up technological convenience, and consider it a worthwhile sacrifice for being part of the Amish community. |
| 4. I sometimes feel as though I am living at the margin of Amish community life. |
| 5. I feel as though following church and community rules restricts my personal desires and values. |
| 6. I feel as though I live comfortably in the lifestyle of the Amish community. |
| 7. Strictly following Amish church and community rules is very important for the mental health of my family. |
| 8. It bothered me that I was unable to continue my formal education beyond 8th grade. |
| 9. Dressing in a similar way to the other members of my Amish community is very comforting for me. |
| 10. I feel as though strictly following Amish church and community rules and standard practices provides comfort and emotional stability for me. |
| 11. It is a stressful choice to not use some modern technological inventions (TV, air conditioner, etc.). |
| 12. I or my family will have to make changes, such as migrating away from my current community; otherwise staying here will be too stressful for us. |
| 13. I have thought very seriously about leaving the Amish community. |
| 14. I feel stigmatized when I interact with people outside of the Amish community. |
| 15. I wish that I could meet more people outside of the Amish community. |

Appendix 1: *Amish Community Stressor Survey initial validation*

First, we assessed test-retest reliability by administering the survey a second time to 20 participants during a planned 2 to 3 year follow-up assessment (average time 2.8 ± 0.4 years, range 2.4-3.6), and found that the scale is reliable with intraclass correlation coefficient (ICC)=0.89, Cronbach’s α = 0.89, p=3x10^-7^. We performed dimension reduction using Principal Component Analysis. An initial unrotated analysis with all 15 questions as components yielded the below Scree plot (figure S1), with the first four components representing 59.2% of variance (table S2).


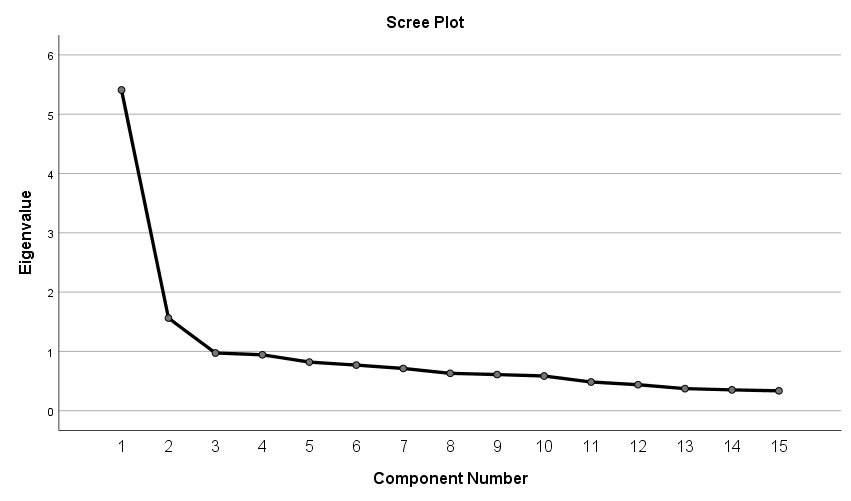


**Figure S1 – Initial dimensional analysis:** Scree plot for initial 15 component analysis.

**Table S2 –** Initial Eigenvalues and extraction sum table for 15-component unrotated PCA model.


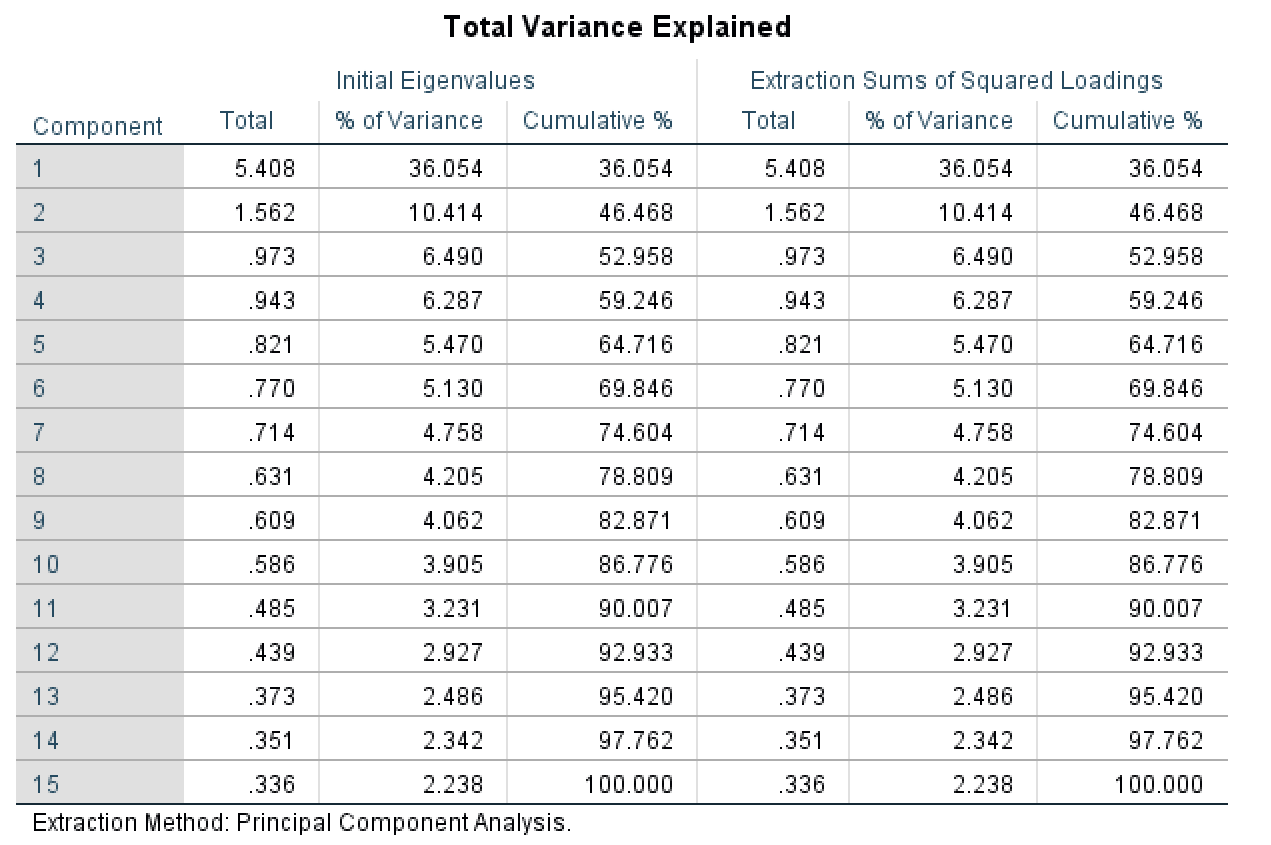


As the 3^rd^ and the 4^th^ components showed eigenvalues right around 1, we proceeded to test three models by including either the first two only, first three only, or all four components, using a Varimax rotation. These models cumulatively explained 46.5%, 53.0% and 59.2% of variance, respectively (Table S3).

We found the four-component model to be the most interpretable with good internal consistency. Component 1 (questions 1, 3, 7, 9 and 10) represents broadly “feeling good to fit in with the community”, Component 2 (questions 2, 4, 5, 6 and 11) represents “stressfulness being among the community”, Component 3 (questions 8, 12 and 15) represents “feeling socially constrained”, and Component 4 (question 14 only) represents “feeling social stigma when interacting outside the community”.

**Table S3 -** Rotated component matrices for the 2, 3, and 4-dimension models (in order, left to right).


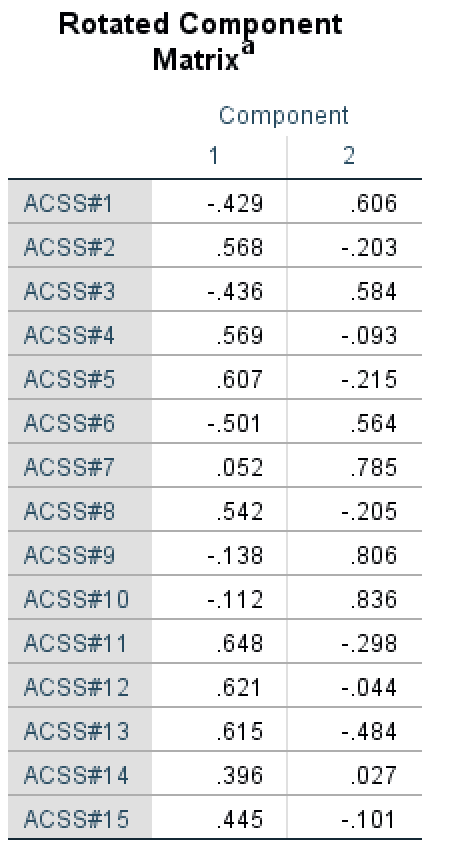

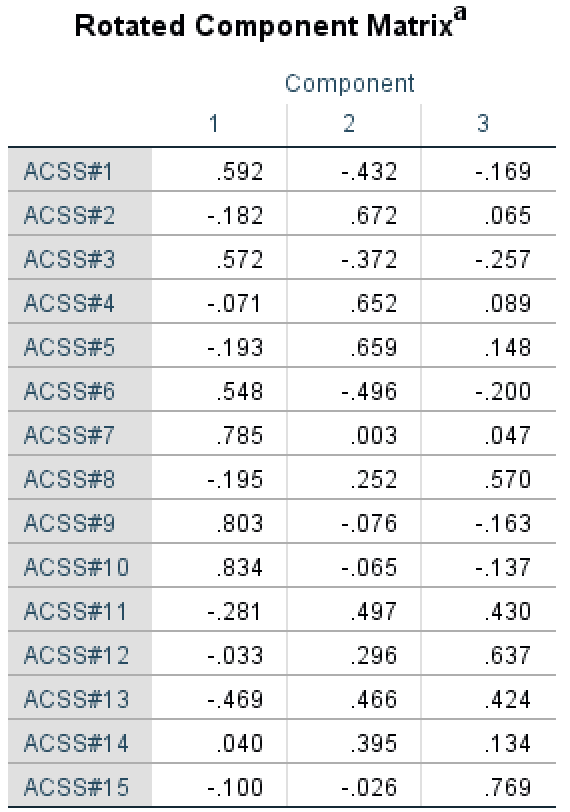

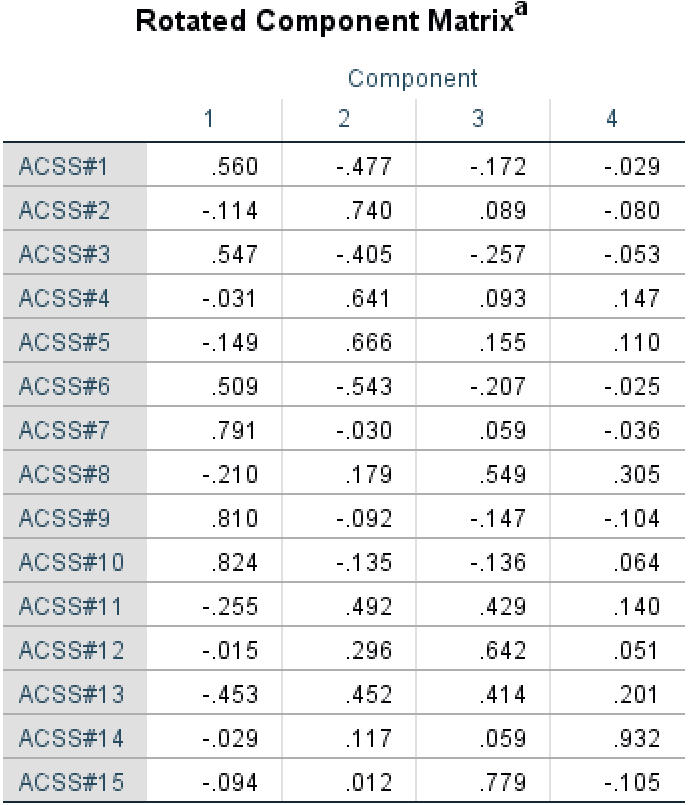


None of the individual components correlated significantly with sex, age, PSS, or number of life stressors. Component 3 was inversely correlated significantly with BDI (r=-0.10, p=0.04). Component 1 was correlated with diagnosis (r=0.10, p=0.04). Correlations between individual components are shown (Table S4). Together, initial validation suggested that this scale demonstrated good reliability and internal consistency and did not substantially recapitulate other individual-level current or lifetime stress measures that we obtained.

**Table S4 – Correlation matrix for 4-dimension model –** Pearson’s r and corresponding *p* value shown for each pair of dimensions, *indicates significance p<0.05/4 as a Bonferroni correction for multiple comparisons

| r  (*p*) | C2 | C3 | C4 |
| --- | --- | --- | --- |
| C1 | 0.47*  *(1.4x10^-25^)* | 0.19* *(9.1x10^-9^)* | 0.004  *(0.9)* |
| C2 |  | 0.49*  *(1.6x10^-27^)* | 0.22*  *(2x10^-6^)* |
| C3 |  |  | 0.23*  *(2x10^-6^)* |
|  |  |  |  |

Amongst all participants, there was a significant inverse correlation between age and level of community-specific stress perception by ACSS (r=-0.19, p=0.0001), but no significant sex effect (t(431)=1.41, p=0.16).
